# Plasmonic Radiation from Spin‐Momentum Locking

**DOI:** 10.1002/advs.202406089

**Published:** 2024-09-02

**Authors:** Yu‐Lu Lei, Juan‐Feng Zhu, Zi‐Wen Zhang, Ji‐Tao Yang, Feng Zhang, Hong‐Sheng Chen, Chao‐Hai Du

**Affiliations:** ^1^ Center for Carbon‐based Electronics School of Electronics Peking University Beijing 100871 China; ^2^ State Key Laboratory of Advanced Optical Communication Systems and Networks School of Electronics Peking University Beijing 100871 China; ^3^ Science, Mathematics, and Technology (SMT) Singapore University of Technology and Design 8 Somapah Road Singapore 487372 Singapore; ^4^ Key Lab. of Advanced Micro/Nano Electronic Devices & Smart Systems of Zhejiang College of Information Science and Electronic Engineering Zhejiang University Hangzhou 310027 China

**Keywords:** chirality, free‐electron radiation, spin‐momentum locking, spoof surface plasmons

## Abstract

Chiral light emission plays a key role in sensing, tomography, quantum communication, among others. Whereas, achieving highly pure, tunable chirality emission across a broad spectrum currently presents significant challenges. Free‐electron radiation emerges as a promising solution to surpass these barriers, especially in hard‐to‐reach regimes. Here, chiral free‐electron radiation is presented by exploiting the spin‐momentum locking (SML) property of spoof surface plasmons (SSPs). When the phase velocity of free electrons matches that of the SSPs, the SSPs can be excited. By implementing wavenumber compensation through perturbations, the confined SSPs are transformed into free‐space free‐electron radiation. Owing to the law of angular momentum conservation, this process converts the transverse spin angular momentum of SSPs into the longitudinal spin angular momentum of free‐electron radiation during the process, producing pure, tunable, and chiral free‐electron radiation across a broad spectrum. This method achieves an optimal degree of circular polarization approaching −1. The innovative methodology can be adapted to SML‐enabled guided states or silicon photonics platforms, offering new avenues for achieving chiral emission.

## Graphical abstract

This study presents a novel method for chiral free‐electron radiation by leveraging spin‐momentum locking (SML) in spoof surface plasmons (SSPs). Through phase‐matching, moving electrons excite SSPs along the modified structure, thus diffracted into Smith‐Purcell radiation (SPR). This approach yields tunable chiral SPR with extensive polarization control and frequency tunability, representing a significant advancement toward achieving near‐unity chiral radiation.

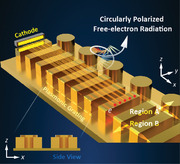

## 1 Introduction

Chirality, the property of asymmetry or handedness, lies at the heart of various phenomena across multiple scientific disciplines and has found numerous applications in diverse fields, including quantum computation,^[^ ^1^ ^]^ holography,^[^ ^2^ ^]^ chiral sensing,^[^ ^3^ ^]^ communication,^[^ ^4^, ^5^ ^]^ and photodetector technology.^[^ ^6^ ^]^ Typically, chiral light emission can be achieved through several methods: chromophores,^[^ ^7^ ^]^ quantum dots,^[^ ^8^ ^]^ metasurfaces,^[^ ^9^ ^]^ photonic crystals,^[^ ^10^ ^]^ and so on. Recent advances in metasurfaces have introduced novel methods for generating chiral emission without spin injection, achieving light emission with a chirality value of 0.98 and a fixed radiation direction by manipulating the topological singularities of quasi‐bound states in the continuum (quasi‐BIC) metasurfaces.^[^ ^11^ ^]^ Additionally, a recent approach involving photonic band BIC metasurfaces, characterized by spin‐valley‐locked features, has reached a chiral purity of 0.91 along with tunable emission angles. However, this approach entails specific design prerequisites and material constraints.^[^ ^12^ ^]^ While these techniques are based on structural resonance, they face challenges related to bandwidth and the inability to adjust the radiation direction finely. In contrast, free‐electron radiation emerges as a compelling candidate for generating chiral radiation, providing a broad tunable frequency range and beam scanning capabilities.

When free electrons interact with photonic structures, free‐electron radiation is induced. It has captivated significant attention for its ability to generate emissions at arbitrary frequency ranges from microwave to X‐ray.^[^ ^13^ ^]^ Smith‐Purcell radiation (SPR) stands out as one of the most explored forms of free‐electron radiation due to its versatility in manipulation. This radiation is generated when an electron moves over a periodic structure, with its emission characteristics determined by the SPR dispersion equation. To date, SPR has found a plethora of applications in various fields such as spectroscopy,^[^ ^14^ ^]^ beam diagnostics,^[^ ^15^, ^16^, ^17^ ^]^ and radiation sources,^[^ ^18^ ^]^ among others. Beyond manipulating various properties,^[^ ^19^, ^20^, ^21^, ^22^, ^23^ ^]^ numerous methodologies have been devised to tailor the polarization, facilitating the generation of chiral SPR. For instance, when the electron interacts with the periodic stacked nanosquare light‐well structures, the chiral SPR is emitted, though the resulting chirality value is limited to 0.45.^[^ ^24^ ^]^ Metasurfaces represent a compelling avenue for exerting control over polarization. Nonetheless, their practical utility is constrained by limitations in bandwidth and structural complexity, which pose significant challenges in achieving tunability.^[^ ^23^, ^24^, ^25^, ^26^, ^27^, ^28^, ^29^, ^30^, ^31^, ^32^, ^33^ ^]^ While the single‐photon source based on nanorods demonstrates the ability for independent and flexible control of spin angular momentum (SAM) and orbital angular momentum, its complex structure poses significant challenges for manufacturing and application.^[^ ^31^ ^]^ Hence, it is crucial to ascertain an efficient approach for generating chiral free‐electron radiation with wide bandwidth, high chirality, and adaptable tunability.

This study introduces a pioneering approach to chiral free‐electron radiation grounded in the principle of spin‐momentum locking (SML) exhibited by spoof surface plasmons (SSPs).^[^ ^34^, ^35^, ^36^ ^]^ Analogous to the behavior of electrons in the quantum spin‐Hall effect, SML involves a universal electromagnetic right‐handed triplet comprising momentum, decay, and spin.^[^ ^37^, ^38^ ^]^ Notably, the propagation direction of the surface wave is locked to its transverse spin angular momentum (T‐SAM). The SML effect in surface waves presents opportunities for directional waveguiding,^[^ ^39^ ^]^ near‐field manipulation,^[^ ^40^ ^]^ and circularly polarized antenna design.^[^ ^41^ ^]^ Theoretical insights elucidate that the SSPs show opposite T‐SAM at different sides of the gratings, revealing this intrinsic characteristic crucial for precisely controlling radiation chirality. SSPs along periodic gratings can be excited by a moving electron through phase‐matching. Precisely introducing perturbations alongside the grating, the SSPs are converted into SPR in the free space via the Brillouin‐folding effect, and the momentum is conversed within this process. The conversion from T‐SAM into longitudinal spin angular momentum (L‐SAM) occurs, giving rise to the chiral SPR emission with customizable polarization.^[^ ^42^ ^]^ Crucially, the spatial arrangement of metallic cylinders relative to the grating is pivotal, which empowers the exploitation of two opposite T‐SAM of SSPs, ultimately bestowing the capability to manipulate the polarization state of SPR. It offers comprehensive control over the polarization state of SPR, spanning left‐handed (LH), right‐handed (RH) circular (with theoretical circular dichroism of −1), and linear polarization. Additionally, the structure provides extensive frequency and direction tunability with a concise design, dramatically easing the tuning and manipulation process. Besides its apparent simplicity, this proposed structure showcases remarkable robustness regardless of frequency regimes and materials. The proposed methodology eliminates the reliance on operation frequency bands and application constraints of traditional lasers and optical polarization elements, enabling the direct production of nearly perfect circularly polarized radiation. With its exceptional radiative characteristics, this innovative structure holds profound potential across diverse applications, encompassing light sources with high chirality and more.

## 2 Results

**Figure 1:**
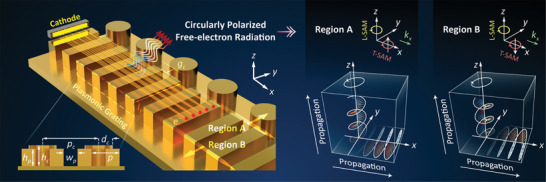
Schematic of Chiral Free‐electron Radiation based on SML. As swift electrons skim over the metallic grating, SML enables the generation of chiral SSPs with opposite T‐SAM, which are then transformed into chiral radiation with opposite L‐SAM based on the Brillouin‐folding effect. The left inset illustrates the structural description of the scheme, while the right insets indicate the chirality of the T‐SAM of SSPs and L‐SAM of SPR. The length, period, depth, and gap width of the metallic grating are *l_p_* = 0.80 mm, *p* = 0.40 mm, *h_p_* = 0.25 mm, and *w_p_* = 0.20 mm. The diameter, period, and depth of the periodically loaded cylinder and its distance away from gratings are *d_c_* = 0.20 mm, *p_c_* = 0.80 mm. *h_c_* = 0.30 mm, *g_c_* = 0.10 mm. The length, width, and thickness of the cathode are denoted as *l_s_* = 0.40 mm, *w_s_* = 0.10 mm, *th_s_* = 0.10 mm.

The schematics of chiral free‐electron radiation are shown in **Figure** **1**. When electrons skim over the metagrating, the interaction gives rise to the generation of SSPs. A pivotal observation here is the behavior of the electric field associated with these SSPs, which demonstrates distinctive spinning actions in two separate regions, identified as A and B. This spinning behavior is central to the chiral nature of the ensuing radiation. By incorporating periodic cylinder array into the structure, the wavenumbers of SSPs are compensated by the modulated period, leading to the rematching of their phase conditions to SPR in the free space. As a result, the tightly confined SSPs, characterized by T‐SAM, are transformed into free‐electron radiation endowed with L‐SAM. As shown in the right insets of Figure 1, the electric field components in region A rotate anticlockwise. Both T‐SAM and L‐SAM display identical rotational characteristics, ensuring the conservation of angular momentum throughout the SSPs to the SPR conversion process. Consequently, circularly polarized SPR is produced. The radiation process in region B behaves similarly, with the only distinction lying in the inverse rotation of the electric field. A comprehensive analysis of this modified structure and its implications will be provided in detail in the following sections.

### 2.1 Spin‐momentum Locking of Spoof Surface Plasmons

Surface plasmon polaritons (SPPs), directing electromagnetic (EM) waves along the dielectric‐metal interface, showcase remarkable proficiency in confining EM fields to this interface in optical regimes.^[^ ^43^, ^44^ ^]^ This remarkably confined surface wave has been validated to manifest the SML phenomenon.^[^ ^36^, ^45^ ^]^ However, at lower frequencies, when the metal behaves as a perfect electric conductor (PEC), conventional SPPs cease to exist. In 2004, a subwavelength textured structure was proposed to support the propagation of surface waves that imitate SPPs at lower frequencies, commonly referred to as SSPs.^[^ ^46^ ^]^ Extensive evidence supports the resemblance of SSPs to SPPs, encompassing aspects such as field confinement and enhancement,^[^ ^47^, ^48^ ^]^ in addition to the manifestation of the SML effect.^[^ ^49^, ^50^ ^]^

To characterize the SML of SSPs, a universal right‐handed triplet comprised of momentum, decay, and spin is introduced.^[^ ^34^ ^]^ The wavenumber of evanescent wave can be expressed as **k *= κ+*** *i* ***η***, where the real part ***κ*** refers to propagation while the imaginary component ***η*** refers to decay. Upon analyzing the dispersion relation in free space, it can be found that **k** satisfies:

(1) $$\left\{\begin{array}{c} \kappa^{2}-\;\eta^{2}=k^{2}\;=k_{0}^{2}\;\\ \kappa \cdot \eta \;=\;0 \end{array}\right.$$

which implies that the decay direction is perpendicular to the propagation direction. The phase delay between propagation and decay is intrinsic to the inherent handedness. Unit vectors $\hat{s}$, $\hat{p}$ are defined to imply the two orthogonal polarizations of the evanescent wave. The vector $\hat{s}$ indicates an *E* field perpendicular to the plane P formed by the vectors ***κ*** and ***η***, while the *E* field lies in the plane P for $\hat{p}$. The basis triplet is chosen as {***κ***, ***η,κ*** × ***η***}. The wavenumber satisfies the transversality condition (**k∙E = 0**) thus the basis is coordinate‐independent. $\hat{s}$ and $\hat{p}$ are independently defined as:

(2) $$\;\hat{s}=\frac{\kappa \times \eta }{\left|\kappa \times \eta \right|}\;=\;i\frac{k\times k^{*}}{\left|k\times k^{*}\right|}$$

(3) $$\hat{p}=\frac{k\times \hat{s}}{\left|k\right|}\;=\;i\frac{k\times \left(k\times k^{*}\right)}{\left|k\right|\left|k\times k^{*}\right|}$$

The field polarization is completely associated with wavevector **k** and this form is so robust that lossy material makes little difference. Inferring from the equations above, we obtain the new form of $\hat{p}$:

(4) $$\hat{p}=\;i\left[\frac{\eta }{\left|k\right|}\left(\frac{\kappa }{\kappa }\right)+i\frac{\kappa }{\left|k\right|}\left(\frac{\eta }{\eta }\right)\right]$$

where $\hat{p}$ is complex and merely a combination of ***κ*** and ***η*** with natural phase quadrature. The polarization state vector $\hat{p}$ is only relevant to the wavevector rather than the specific value of electric fields. Thus, there exists intrinsic polarization which is decided by wavenumber **k**. The spin vector $\hat{s}$ is orthogonal to ***κ*** and ***η***, which are closely relevant with $\hat{p}$. With decay ***η*** fixed, the inverse propagation −***κ*** will undoubtedly result in the reversed rotation of $\hat{p}$ and flipped $\hat{s}$. It demonstrates that the spin is locked to the propagation, which is interpreted as “spin‐momentum locking” with a triplet of propagation (***κ***), decay (***η***), and spin ($\hat{s}$).

It can be also proved from another perspective. Concerning a 2D system independent of the *y*‐direction, the propagating surface wave of the electric field in the plane perpendicular to the interface can be expressed as:^[^ ^51^ ^]^

(5) $$\left\{\begin{array}{c} \;E_{x}=\;A\frac{\sqrt{k_{x}^{2}-k_{0}^{2}}}{\omega \varepsilon_{0}}e^{-ik_{x}x}e^{-\sqrt{k_{x}^{2}-k_{0}^{2}}z}\\ \;E_{y}=\;0\\ \;E_{z}=\;iA\frac{k_{x}}{\omega \varepsilon_{0}}e^{-ik_{x}x}e^{-\sqrt{k_{x}^{2}-k_{0}^{2}}z} \end{array}\right.$$

It can be inferred from Equation (5) that the SSPs are transverse magnetic polarized and the component *E_x_* is perpendicular to *E_z_*. Given that *k_x_* > *k_0_*, where each parameter represents the wave vector outside the structure, *k_z_* should be imaginary since $k_{x}^{2}+\;k_{z}^{2}=k_{0}^{2}$. Consequently, a distinct 90° phase shift exists between *E_x_* and *E_z_* in the *x‐z* plane, affirming its spin‐orbit property. Furthermore, for a system with finite length along the *y*‐direction, the wave vector relation is expressed as $k_{x}^{2}+k_{y}^{2}+\;k_{z}^{2}=k_{0}^{2}$. Since *k_x_* > *k_0_*, *k_y_*, and *k_z_* are imaginary because of the attenuation of field components in the transverse plane of the SSPs waveguide. In this scenario, an additional 90° phase shift exists between the *E_x_* and *E_y_* field components. In conclusion, these chiral SSPs are conclusively demonstrated to possess spin‐orbit properties in both the *x‐y* plane and the *x‐z* plane.^[^ ^41^ ^]^

To analyze the structure theoretically, it is imperative to study the dispersion relation. The dispersion equation within the boundaries of the PEC can be expressed as:^[^ ^52^ ^]^

(6) $$\frac{1}{k_{zn}}\sum_{-\infty }^{\infty }Sa^{2}\;\left(\frac{k_{xn}w_{p}}{2}\right)=\frac{p}{w_{p}}\;\cot\left(k_{0}h_{p}\right)$$

where *k_xn_ = k_x0_+2πn/p* stands for the longitudinal wavenumber of the *n^th^* harmonic mode and $k_{zn}=\sqrt{k_{0}^{2}-k_{xn}^{2}}$.

**Figure 2:**
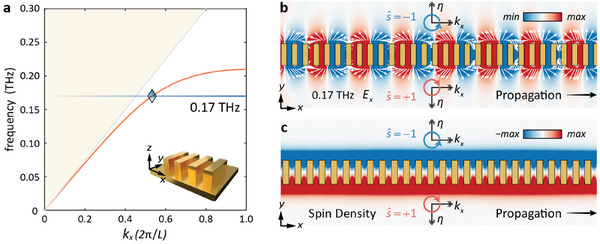
The theoretic analysis of SSPs. a) Dispersion curve of SSPs. b) Electric field distributions of SSPs. c) Spin momentum locking phenomenon.

To validate the proposed theory, a model is constructed using COMSOL and subjected to analysis. **Figure** **2**a illustrates that SSPs serve as slow wave modes, characterized by a dispersion curve that lies below the light line. As the wavenumber increases, the stimulated SSPs gradually localize onto the surface. Conventional research mainly focuses on the strong field confinement as well as the electric field components in the *x‐z* plane with L‐SAM in the *x*‐direction while few delved into the *x‐y* plane. In this study, the T‐SAM in the *x‐y* plane will be harnessed to generate circularly polarized radiation. The electric field distribution at 0.17 THz is depicted in Figure 2b with white arrows denoting the counter‐rotating direction of the electric field. The density of SAM is quantified by the following expression:^[^ ^53^, ^54^ ^]^

(7) S=14ωImεE∗×E+μH∗×H

When *S* < 0, the electric components spin anticlockwise, resembling the rotational movement of the spokes of a rolling bicycle wheel.^[^ ^55^ ^]^ The unit spin vector is calculated theoretically as $\hat{s}=\;-1$, indicating its RH polarization. Similarly, the electric field rotates inversely when *S >* 0 with $\hat{s}=\;+1$, corresponding to LH polarization. The normalized SAM density based on the total energy has been portrayed in Figure 2c. The analysis of SAM is found to align with the simulation results, thereby affirming the correctness and validity of the proposed theory.

### 2.2 Chiral Free Electron Radiation Based on SML

The preceding discussions have confirmed the existence of SML carried by the SSPs. Efforts will then be attributed to how to utilize this property and generate chiral SPR. As the emitted electrons skim over the gratings, the SSPs are stimulated and propagate along the periodic structure. To harness the spin‐orbit characteristic of the SSPs, periodic components or perturbations are incorporated into the modified structure, which originally comprises metagrating. These introduced modifications serve to convert the highly confined surface wave into a radiative wave via the Brillouin‐zone folding effect.^[^ ^56^, ^57^ ^]^

**Figure 3:**
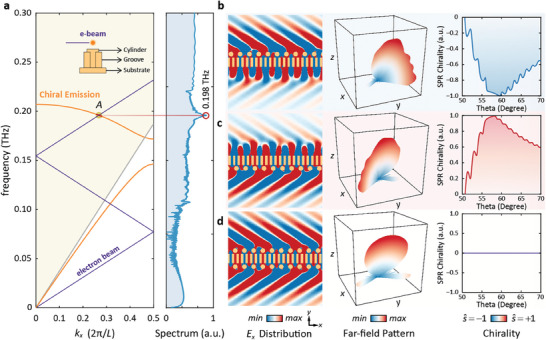
Chiral SPR emission. a) Dispersion diagram of the interaction system. The orange and purple lines represent the dispersion curves of the modified structure and the electron beam, respectively. b–d) Electric field distribution in the *x–y* plane, far‐field pattern, and chirality with cylinders on *+y* side (b), *−y* side (c), and both sides (d).

The dispersion curve of incident electrons can be expressed as:

(8) $$\;\omega_{e}=v_{e}\;\;k_{x}=\;\beta ck_{x}$$

where *v_e_* represents the velocity of the electrons, and *β = v_e_/c* indicates the normalized electron velocity. As depicted in **Figure** **3**a, the orange solid line signifies the dispersion curve of the chiral SSPs while the purple folded line denotes the electron dispersion. Compared with the curve in Figure 2a, the dispersion curve of the modified structure is folded twice due to the doubling of the structure period. This can be explained that with different periods, the Brillouin zone is reshaped based on the Brillouin folding effect.^[^ ^42^ ^]^ Moreover, the partial curve of SSPs lies above the light cone, symbolized by the gray line, indicating the presence of resonant SSPs. These resonant SSPs are readily transformed into enhanced SPR since their wavevectors are matched with those of the free space. Hence, upon excitation by swift electrons, as illustrated by the intersection of the two curves, the chiral free‐electron radiation initially manifests as evanescent waves carrying T‐SAM and then diffracted into chiral SPR carrying L‐SAM. Take the intersection point in Figure 3a as an example, the operating frequency is identified at 0.198 THz, consistent with the results obtained from the radiation spectrum in the simulation in the right inset.

To further investigate the polarization states of generated SPR, the model is modeled in Particle in Cell solver in CST STUDIO SUITE. Copper with a conductivity equal to 5e7 S m^−1^ is employed to construct the structure. In the initial configuration, the cylinders are set in the *+y*‐direction as indicated by Region A in Figure 1, where the electric field component is rotating anticlockwise. Through the influence induced by the cylinders, the T‐SAM of SSPs is transformed into L‐SAM carried by SPR. As portrayed in the first column of Figure 3b, the electric field in the +*y*‐direction is noticeably stronger than that on the other side, indicating the contribution of the added cylinders to the generation of SPR. Moreover, the corresponding far‐field pattern is depicted in the second column, from which the radiation pattern can be explicitly observed. The predominant radiation lobe in the *z*‐direction is attributed to the SPR carrying L‐SAM, showcasing remarkable chirality. The other comparatively strong lobe in the *x‐y* plane can be interpreted as the surface wave coupled out by the added cylinders.

The degree of circular polarization (DCP) is employed as a metric to quantitatively assess the spin degree of the radiation:^[^ ^58^ ^]^

(9) $$\mathrm{DCP}\;=\frac{I_{L}-I_{R}}{I_{L}+I_{R}}$$

where *I_L_* and *I_R_* separately render the intensity of the LH and RH components of the electric field. Due to the strong confinement of SSPs, the zeroth wavenumber (*k_x0_*) in the propagation direction significantly exceeds the wavenumber in the free space (*k_0_*). Conversely, the wavenumbers of higher harmonics (*k_xn_*) can be effectively coupled out. Assuming an angle *θ* between the main lobe in the *z*‐direction and the *x*‐axis, the relationship describing the radiation angle to the direction of electron movement can be described as follows:^[^ ^52^ ^]^

(10) $$k_{x0}+\;\frac{2n\pi }{L}=k_{0}\;\cos\theta$$

The analysis curve of the DCP in the third column of Figure 3b reveals that SPR generated in the structure exhibits right‐handed circular polarization (RCP), specifically in the primary direction at an angle of 58°, which also conforms to the SPR dispersion relation as expressed in Equation (10) (*L* = 2*p*, *n* = −1). The closer the DCP value approaches −1, the purer the RCP is. The achieved optimal value is −0.997, signifying an almost perfect RCP.

Similarly, as shown in Figure 3c, when the cylinders are positioned in the −*y*‐direction where the electric field components rotate clockwise, the transformation process of SSPs from surface wave into radiation is quite evident according to the electric field distribution in the *x‐y* plane. Consequently, the generation of SPR can be confirmed. This conclusion is further supported by the far‐field pattern illustrated in the second column. The main lobe, bearing L‐SAM, propagates along the *z*‐direction, ascribed to the generation of SPR. Meanwhile, the side lobe in the −*y* direction arises from the surface wave coupled out by the external cylinders. Regarding chirality, the DCP values consistently exceed 0, indicating the predominance of LH polarization. In the main direction of 58°, the ideal DCP value equals +0.992, signifying a perfect left‐handed circular polarization (LCP).

Furthermore, when cylinders are present on both sides as portrayed in Figure 3d, a linearly polarized (LP) SPR emerges due to the synchronization of two opposing circularly polarized waves. In this configuration, the electric field intensity is remarkably consistent on both sides of the structure. As depicted in the second column of Figure 3d, the main lobe appears in the *z*‐direction with an angle of 57.5°, demonstrating the presence of SPR in the free space with two side lobes in the *x‐y* plane, caused by the addition of the two arrays of cylinders. Importantly, the DCP remains 0 across all angles, providing further evidence for the presence of LP SPR in this system.

Additionally, the electric field distribution in the *y‐z* plane, as well as the *x‐z* plane, can also serve as evidence for the generation of SPR (see detailed information in Section S1, Supporting Information). It can be found that the tightly bound surface wave can be effectively coupled out on the side with the accessional cylinders in the form of SPR. With perturbations on both sides, the radiation will propagate along the *+z*‐direction straightforwardly. The three electric field components are shown in the third column independently along with the direction of SPR, which is aligned with the theoretic analysis. Also, the far‐field patterns under three independent circumstances from another perspective are delineated in the last column and facilitate a better understanding of the radiation behaviors of the SPR. The successful generation and polarization manipulation of SPR have been validated and further confirmed through simulations. Introducing the modified periodic structure enables convenient control over the polarization state of SPR. These findings open up new possibilities for utilizing SPR in various novel applications, particularly in the fields of detection and communication.

## 3 Discussion

**Figure 4:**
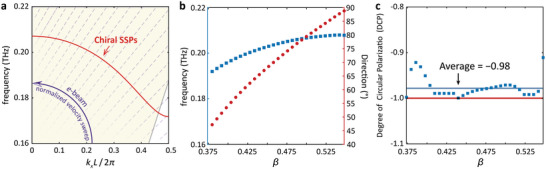
Tunability analysis of modified chiral SPR with normalized beam velocity varying a) theoretical operation frequency; b) simulation frequency and main lobe direction; c) degree of circular polarization analysis.

The analysis presented so far has focused on a single operating frequency. However, by adjusting the energy of the electrons, both the operating frequency and the direction of SPR can also be tuned over a wide spectral range. Without loss of generality, the discussions in the following are performed based on the structure that generates RCP SPR. As illustrated in **Figure** **4**a, the red solid curve corresponds to the resonant SSPs while the purple dashed lines depict the electron dispersions with different velocities. Notably, as the normalized electron velocity increases, the wave vector as well as the group velocity of the stimulated SSPs decrease gradually, along with a slower shift in the operation frequency. Simultaneously, the difference between the direction of radiation diffracted from the compound gratings and the propagation direction of the SSPs steadily expands.

To confirm the validity of the prediction, a sequence of simulation experiments was meticulously conducted, systematically adjusting the normalized electron velocity. The results are depicted in Figure 4b,c. As *β* rises from 0.35 to 0.55, the interaction frequency shifts from 0.19 THz to 0.21 THz, aligning precisely with the theoretical analysis. Furthermore, the radiation angle undergoes a transition from 45° to 90°, clearly demonstrating the beam‐scanning capability. Notably, when the wave vector reaches 0, the free‐electron radiation emission occurs perpendicular to the gratings, with a radiation angle of 90°.

As portrayed in Figure 4c, the DCP of the emitted chiral SPR is also meticulously examined by tuning the electron velocity, revealing a remarkable average DCP value of ≈−0.98, with the optimal value approaching −1. This compelling observation serves as convincing evidence, demonstrating the exceptional capability of the proposed methodology in achieving extremely chiral radiation. Furthermore, the consistent RCP nature of the generated SPR, even with variations in normalized beam velocity ranging from 0.35 to 0.55, further underscores the stability and versatility of the system.

So far, the demonstrated structure allows for the generation of controllable chiral SPR with wide tunability in both direction and frequency by simply adjusting the normalized electron velocity. Moreover, the emitted SPR remains great chirality with an average DCP equal to −0.98 when the beam velocity is altered. The robustness of the radiative properties is protected by the inherent nature of SML. Even when the structure is assigned as either PEC or dielectric with higher permittivity, the performance remains almost unaffected.

Furthermore, we have also investigated several methods to generate polarization‐controllable radiation and their performances. Refs. [12, 26] independently introduce metasurfaces as a means to control the emission properties. Nevertheless, both inevitably face challenges including narrow bandwidth and difficulties in tuning due to the complicated configuration. Moreover, their radiation direction cannot be tuned continuously. Ref. [24] demonstrates the generation of chiral SPR, in which the polarization state is manipulated by electron beam injection. However, its chirality is limited and <0.45. In comparison to these studies, our proposed method offers several advantages. Since the methodology is rooted in the transient nature of SSPs and the handedness of the evanescent wave remains undisturbed, the generated SPR demonstrates an extraordinary chirality value of up to 1. In contrast to the tuning difficulties posed by structure complexity in metasurfaces, the modified structure, comprising metallic gratings and cylinders, provides a simple approach for beam manipulation and polarization control. Additionally, by tuning the electron velocity, the method exhibits beam‐scanning capability across a wide frequency range. Leveraging the SML principle, an intrinsic property of SSPs, this methodology is universally established regardless of materials or frequency. Its versatility spans from microwave to hard‐to‐reach regimes, including X‐ray and far‐infrared frequencies, emphasizing its exceptional robustness. This approach presents a promising solution for the development of compact on‐chip THz radiation sources capable of directly coupling out the emitted radiation.

## 4 Experimental Validation

**Figure 5:**
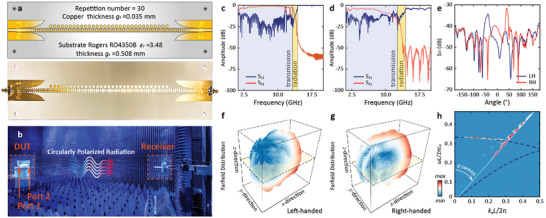
Experimental verification. a) Conceptual and fabricated antenna in the microwave band. b) Experimental environment and setup for testing the microwave antenna. c,d) *S* parameters for SML‐based microwave antenna presented in both simulation and experimental results. e) Radiation pattern of the radiation. f,g) Far‐field radiation plot for LH and RH components and the light‐yellow plane stands for the modified structure. h) Measured evolutions of the S_21_ spectra versus radiation angle *φ* where *φ* is remapped into wavenumber space. The dark blue dashed line represents the theoretical dispersion analysis.

Notably, the SML‐based method is not only robust in 3D gratings but also extends its applicability to 2D printed structures. As the limited experimental setup, we fabricated a planar antenna and conducted tests in the microwave regime to experimentally validate the underlying physics. The prototype is depicted in **Figure** **5**a, which is constructed on a Rogers RO4350B substrate with a relative permittivity of 𝜀_𝑟_ = 3.48 along with its conceptual design (see detailed information in Section S2, Supporting Information). The substrate has a thickness of 0.508 mm, and the printed metallic structure is 0.035 mm thick. The repetition number for the unit cell, consisting of one circular patch and two gratings, is set at 30, ensuring adequate support for the generation of SSPs. Since manipulating the electron beam in experiments is challenging, the TEM mode of the coplanar waveguide is considered as equivalent electrons, with a prerequisite of a perfect match between the transmission line and SSPs waveguide. The matching bridge with gradient grooves and flaring ground facilitates the conversion of guided waves into SSPs.^[^ ^59^ ^]^ Moreover, the input port impedance is optimized to 50 Ω, enabling direct SSPs excitation through a transmission line and allowing verification of functions such as DCP and beam‐scanning capability.

The experimental setup occurs within a microwave anechoic chamber, as illustrated in Figure 5b. The device under test (DUT) is horizontally fixed on the platform, nearly aligning with the high‐gain horn antenna for signal reception and far‐field radiation performance measurement. The fabricated antenna is connected to a vector network analyzer (VNA) via an N‐type connector and powered through the subminiature version A (SMA) port throughout the tests. Port 1 of the printed antenna links to the VNA via the SMA port, while Port 2 connects to a matching load. The highly circularly polarized radiation will then propagate and be received by the receiver antenna.

The simulated S parameters of the planar prototype are depicted in Figure 5c. The incorporation of circular patches unveils a radiation window when reflection parameter S_11_ and transmission parameter S_21_ are both relatively low, encompassing the frequency range of ≈13.3–14.2 GHz, delineated by the highlighted yellow region in the figure. Subsequent experimentation involves the modified structure, and the experimentally derived S parameters are showcased in Figure 5d, demonstrating a high consistency with the trend demonstrated by the simulation except for the radiation window ranging from 12.6 to 14.0 GHz. The radiation properties at 13.6 GHz are then studied in detail. The E‐field radiation pattern of LH and RH components are shown in Figure 5e, while the 3D far‐field patterns for each component are illustrated in Figure 5f,g respectively. The observed separation of linear guided waves into two circularly polarized radiations with a high DCP is denoted by the light‐yellow plane which showcases the structure plane, achieving a perfect RCP with a DCP of −0.997.

Furthermore, the dispersion spectrum, shown in Figure 5h, is obtained by adjusting the angle *φ* between the receiver and DUT. The theoretic dispersion curve of the modified microwave antenna is presented by the dark blue dashed line. Clear peaks inside the light cone align thoroughly with the theoretic analysis, showcasing the beam‐scanning function of the prototype. This not only establishes a solid foundation for its feasibility but also emphasizes the robustness and generality of its underlying principle. These observations suggest potential applications in other frequency regimes, such as the THz band.

## 5 Conclusion

In this investigation, the methodology for achieving chiral free‐electron radiation is proposed by merit of SML of evanescent waves. Leveraging the Brillouin‐zone folding effect, the SSPs carrying T‐SAM are converted into chiral SPR carrying L‐SAM, all while preserving the rotating direction. The precise arrangement of the perturbations and gratings critically influences the polarization state of the emitted radiation. Consequently, the proposed scheme enables the independent generation of RCP, LCP, and LP SPR. Moreover, by simply tuning the beam velocity, remarkable tunability in terms of frequency and direction is achieved while maintaining a high degree of circular polarization throughout the process. A prototype antenna, fabricated and tested in the microwave regime, demonstrates high DCP based on the SML principle. Radiation pattern and DCP have been independently confirmed, aligning well with theoretical analysis. This experimental validation underscores the feasibility of the proposed scheme, with potential extension into the THz band. Overall, this study presents a promising pathway for polarization‐controllable chiral free‐electron radiation, offering new prospects for the design and implementation of advanced compact chiral THz radiation sources, which may find a plethora of applications in diverse fields such as detection, communication, chiral sensing, quantum computation, particle detection, and energy transmission.

## Conflict of Interest

The authors declare no conflict of interest.

## Author Contributions

Y.‐L.L. and J.‐F.Z. contributed equally to this work. Y.‐L.L. conceptualized the idea for the study; designed the methodology; made the software; performed validation and visualization; wrote the original draft; and reviewed and edited the manuscript. J.‐F.Z. conceptualized the idea for the study; performed data curation; designed the methodology; performed validation; wrote the original draft; and reviewed and edited the manuscript. Z.‐W.Z. conceptualized the idea for the study; designed the methodology; performed validation and visualization; wrote the original draft; and reviewed and edited the manuscript. J.‐T.Y. performed data curation, investigation, and software, and reviewed and edited the manuscript. F.Z. performed data curation, investigation, and software, and reviewed and edited the manuscript. H.‐S.C. performed project administration and supervision. C.‐H.D. conceptualized the idea for the study; performed funding acquisition, project administration, and supervision; and reviewed and edited the manuscript.

## Supporting information

Supporting Information

## Data Availability

The data that support the findings of this study are available from the corresponding author upon reasonable request.
