# Supplementary material for: Plasmonic Radiation from Spin‐Momentum Locking
Source: Adv Sci (Weinh). 2024 Sep 2;11(40):2406089. doi: 10.1002/advs.202406089 (PMC11515908; doi:10.1002/advs.202406089)
Supplement: Supplementary file 1 — Supporting Information [file ADVS-11-2406089-s001.pdf]

ADVANCED SCIENCE
Open Access

Supporting Information

for *Adv. Sci.*, DOI 10.1002/advs.202406089

Plasmonic Radiation from Spin-Momentum Locking

*Yu-Lu Lei, Juan-Feng Zhu, Zi-Wen Zhang, Ji-Tao Yang, Feng Zhang, Hong-Sheng Chen and Chao-Hai Du**

Supporting Information for Plasmonic Radiation from Spin-Momentum Locking

*Yu-Lu Lei, Juan-Feng Zhu, Zi-Wen Zhang, Ji-Tao Yang, Feng Zhang, Hong-Sheng Chen, and Chao-Hai Du**

Table of contents

S1. Electric field distribution and far-field pattern of the modified structures

S2. Detailed information on the microwave experiment

S1. Introduction Electric field distribution and far-field pattern of the modified structures.

As shown in Figure 3 in the manuscript, a set of cylinders can be leveraged to couple out the tightly confined SSPs. The evanescent wave carrying T-SAM, whose chirality is solely dependent on the direction of propagation and decay, undergoes the transformation into SPR, which bears L-SAM with the same chirality. The electric field distribution under three different structure configurations will be depicted and discussed in detail.

As depicted in **Figure S1** (a), the cylinder perturbations are loaded on the $+y$ side with the unit cell consisting of two gratings with a single cylinder. Leveraging the SML principle, the electric field components rotate anticlockwise on the $+y$ side to the metagrating, which can be further characterized as T-SAM = −1. Once the cylinders are introduced, the wavenumber of tightly confined surface waves, referred to as SSPs, is compensated to match that of the free space. Consequently, the SSPs will be coupled out and transformed into SPR carrying L-SAM which share the same chirality with T-SAM. Apparently, the E_x component in the $+y$ direction is much stronger than the other side in the x-y plane. When it comes to the y-z plane, the E_x component tends to be biased towards the side with the additional structure, which is the $+y$ side under this configuration. The three components in the x-z plane, encompassing E_x, E_y, and E_z, are independently portrayed in the third column of Figure S1 (a) and corroborate the radiation generation. The E_x component radiates into free space with the assistance of cylinders and the E_z component exhibits nearly the same strength. The radiation angle to the structure plane can be observed to be 58°. The last row shows another perspective of the far-field pattern of the modified structure, from which the radiation pattern can be observed more intuitively. The main lobe along the $+z$ direction is interpreted as SPR showcasing exceptional chirality while the side lobe in the x-y plane is caused by the surface wave coupled out by the cylinders.

Similarly, when the cylinders are attributed on the $-y$ side, the surface wave is transformed into SPR with an opposite handedness, as the electric field components rotate in the opposite direction compared with the situation above according to the SML principle. The field density of the E_x component with cylinders is noticeably greater than the other side as shown in the first image of Figure S1 (b). Additionally, in the y-z plane, the radiation shows a tendency to bias towards the perturbations, aligning with the discussion. The field distributions in the x-z plane with an angle of 58° to the $+x$ direction further confirm the generation of SPR. The far-filed pattern, viewed from a different angle, offers a deeper insight.

In the scenario with two sets of cylinders positioned beside the gratings, the additional structures facilitate the coupling-out process of the SSPs, leading to a noticeable field distribution on both sides, as illustrated in the first figure in Figure S1 (c). Due to the synchronization of two opposite circularly polarized waves, the result turns out to be a linearly polarized radiation propagating straight along the $+z$ direction. In the x-z plane, the electric components E_x and E_z behave similarly to the illustration above. However, the E_y component vanishes because of synchronizing the two opposite circular polarizations. The radiation aims at 57.5° deviating from the $+x$ direction. The portrait of the far-field pattern still exhibits the main lobe in the $+z$ direction and two side lobes in the structure plane, caused by the SSPs'

transformation.

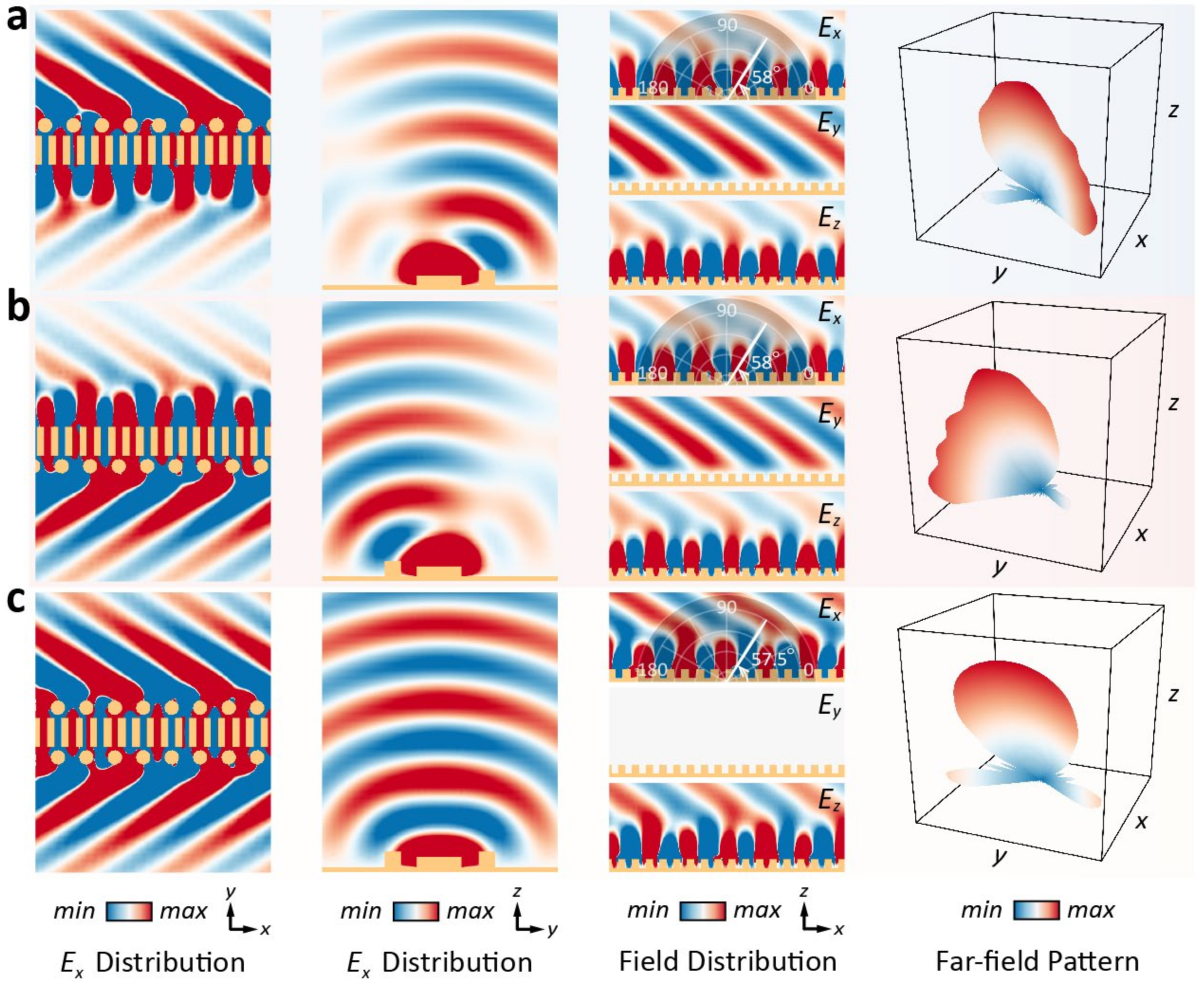

Figure S1. Electric field distribution and far-field pattern under different configurations (a) With cylinders on the +y side; (b) With cylinders on the −y side; (c) With cylinders on both sides.

S2. Detailed information on the microwave experiment

At the outset, it's imperative to ascertain the manifestation of spin-momentum locking (SML) within the engineered planar antenna, comprised of metallic gratings and cylinder patches. This phenomenon dictates that once the propagation direction of the evanescent wave within the structure is established, the rotation of the electric components adjacent to the gratings adheres uniquely to the SML principle, thereby showcasing exceptional circular polarization purity.[1] As shown in **Figure S2** (a), upon the passage of the e-beam over the metallic gratings, the excitation of SSPs occurs, which are subsequently confined to the surface of the structure. Owing to the SML principle, the electric field component in Region A undergoes an anticlockwise rotation, attributed to its attenuation along the $+y$ direction, equating to T-SAM = −1. Conversely, the electric field within Region B experiences a reverse rotation due to its opposite decaying direction, resulting in T-SAM = +1.

To experimentally validate the proposed scheme, a corresponding structure operating in the microwave band was fabricated. The dimensions of the metallic gratings, including length (l_p),

period (p), depth (h_p), and gap width (w_p), are set to $l_p = 7.00$ mm, $p = 3.50$ mm, $h_p = 2.80$ mm, and $w_p = 1.40$ mm. Subsequently, the structure was meticulously constructed within CST STUDIO SUITE, and the resulting field distribution is depicted in Figure S2 (b). The theoretical demonstration of the normalized spin density distribution, as delineated by Equation (7) in the manuscript, reveals exceptionally high purity within Regions A and B upon independent scrutiny. Furthermore, the introduction of cylinder patches yields negligible alteration to the rotation behavior of the electric field, as evidenced by Figure S2 (c), wherein the rotational direction remains unchanged. Validation of the modified structure within commercial software corroborates the persistence of a high degree of spin purity on both sides, as illustrated in Figure S2 (d).

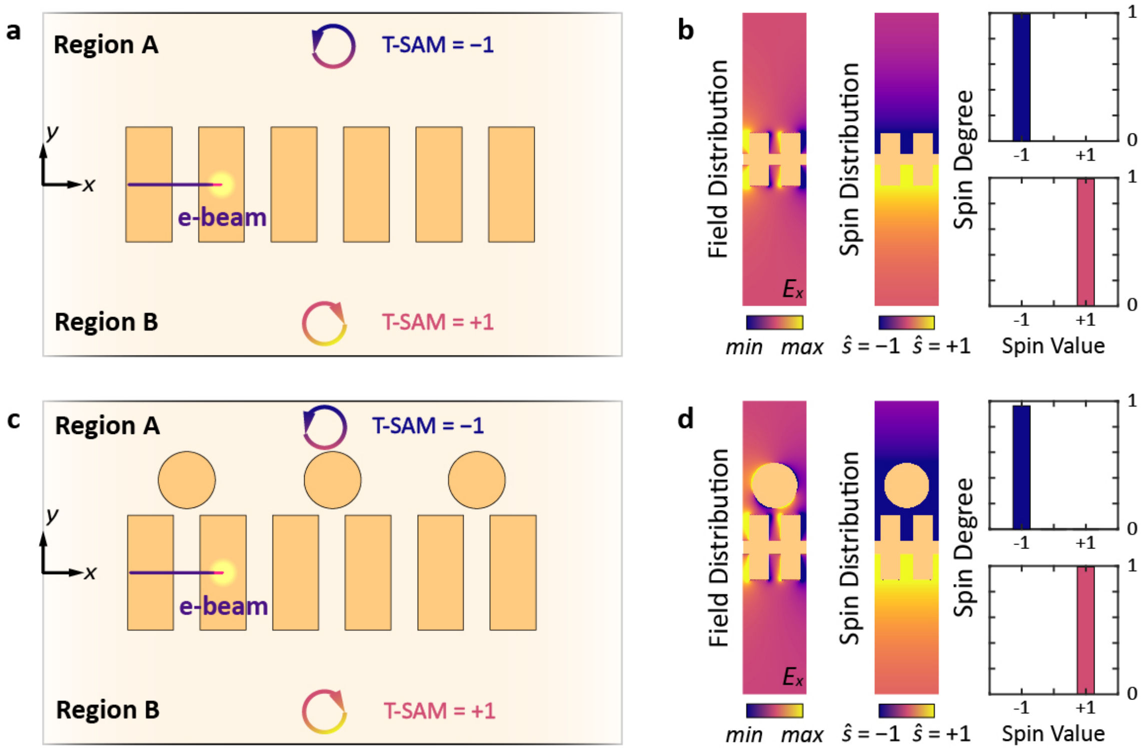

Figure S2. Schematic diagram along with field distribution, theoretic spin distribution, and spin degree for each structure. (a) and (b) are for the metallic gratings, while (c) and (d) stand for the modified structure.

In the experimental setup, since it's quite challenging to manipulate the moving electrons, the TEM mode of CPW will be leveraged to excite confined SSPs on the gratings efficiently, facilitated by a high-efficiency broadband conversion structure illustrated in **Figure S3** (a). This unique section establishes a seamless transition between the conventional guided wave TEM mode in the CPW segment and the SSPs confined to the periodic section, achieved through the incorporation of a tapering section.[2] Part 1 of the structure, with a length of 25 mm and a ground width (W_g) of 25 mm, is optimized to meet the requirement for 50 Ω impedance. The input port of the CPW segment, depicted in Figure S3 (b), is meticulously designed with a central conductor width (C_i) of 5 mm and a gap between the central conductor and the ground (C_g) of 0.26 mm. Additionally, the conductor width (C_0) becomes 7 mm to connect to Part 2. Part 2 constitutes a tapering section spanning 28 mm, comprised of 8 grooves with increasing depth. It serves to match the momentum of the conventional CPW to the SSP waveguide. As illustrated in Figure S3 (c), the deeper the groove, the lower the asymptotic frequency,

culminating in a dispersion curve serving as a theoretical depiction of the proposed SSP waveguide. These curves reside to the right of the light cone, indicative of its slow-wave characteristics.

Then the structure is meticulously constructed in the CST and the field distributions of the E_x component in the x-y plane are depicted in Figure S3 (d) and (e). As illustrated in Figure S3 (d), successful conversion of the guided wave into confined SSPs within the plasmonic waveguide is achieved. Upon integration of cylinder patches, the surface wave is effectively coupled out, leading to the formation of resonant SSPs, as evidenced in Figure S3 (e), where the wavenumber is suitably compensated. In summary, the proposed high-efficiency conversion structure exhibits proficiency in exciting SSPs, a fact substantiated by the simulation results.

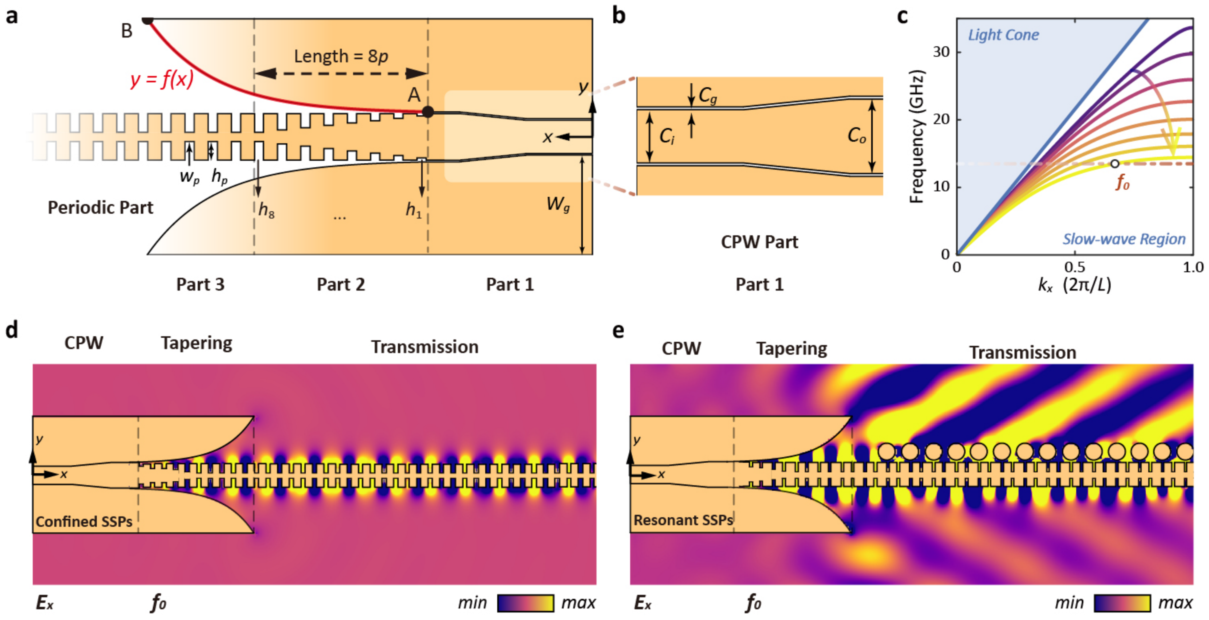

Figure S3. Illustration of the microwave antenna along with the electric field distribution. (a) Schematic diagram of the CPW-plasmonic waveguide; (b) The CPW part; (c) Dispersion curves of metallic gratings with varying depths printed on the substrate; (d) Electric field distribution of Ex of the metallic gratings; (e) Electric field distribution of Ex of the proposed structure.

The subsequent experimental phase is conducted, as delineated in **Figure S4** (a). The DUT is positioned atop a rotating platform within a microwave anechoic chamber, enveloped by absorptive materials to facilitate far-field data acquisition. The receiver antenna, nearly matching the height of the DUT, is deployed to capture radiation. Port 1 of the DUT is connected to an Agilent N5245A via an N-type connector, powered through the SMA port during the entirety of the tests, while Port 2 interfaces with a matching load. Subsequently, the acquired data is exported and processed on a laptop.

The actual experimental setup is depicted in Figure S4 (b), comprising the DUT, VNA, receiver horn antenna, and turntable. The fabricated structure is situated on the turntable, which aligns with the height of the receiver horn antenna. The turntable facilitates rotation, enabling signal reception from diverse directions by the receiver. The receiver is connected to the VNA through a coaxial transmission line. Apart from the far-field test, a near-field test is conducted, as illustrated in Figure S4 (c). All setups remain consistent, except for employing a smaller type of receiver to capture more detailed information. The footprint of the receiver horn antenna is

significantly smaller compared to the DUT, ensuring the precision of the conducted experiment. The far-field pattern of the proposed antenna is separately acquired and processed for left-handed and right-handed components. At 13.6 GHz, the DCP value for the proposed antenna attains −0.997, indicating nearly perfect right-handed circular polarization.

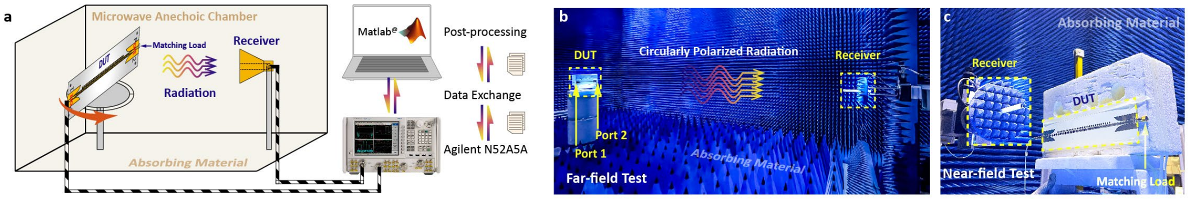

Figure S4. Illustration of the experiment. (a) Schematic illustration of the experimental setup; (b) Real experimental setup in the far-field test; (c) Real experimental setup in the near-field test.

The aforementioned experiment serves to validate the generation of circular polarization radiation with high purity. Subsequently, the beam-scanning capability of this structure will be scrutinized for validation through the following experiment. The schematic illustration of the experimental setup is depicted in **Figure S5** (a). The DUT is vertically positioned on the turntable, while the receiver horn is situated on the rack. The horn rotates around the DUT to receive radiation from various directions. The two CPW parts of the DUT independently connect to ports 1 and 3 of the VNA via coaxial transmission lines, while port 2 of the VNA connects to the receiver to obtain the far-field pattern of the manufactured leaky-wave antenna. Subsequently, the acquired data is selectively exported and processed.

The actual experimental setups are illustrated in Figure S5 (b), wherein the foam board is utilized to mount the antenna vertically on the turntable. In the setup, the receiver is affixed to the arm of a single-arm turntable for capturing radiation signals. The sliding track on the arm facilitates adjustment of the distance between the DUT and the receiver. Subsequently, the receiver rotates around the DUT, enabling multifaceted acquisition of outwardly radiated signals. The acquired data undergoes post-processing to derive the dispersion spectrum, obtained by adjusting the angle φ between the receiver and DUT, as illustrated in Figure S5 (c). The resonant peaks exhibit remarkable consistency with the theoretical dispersion line represented by the black dotted line. Furthermore, all peaks fall within the light cone, signifying their ability to radiate.

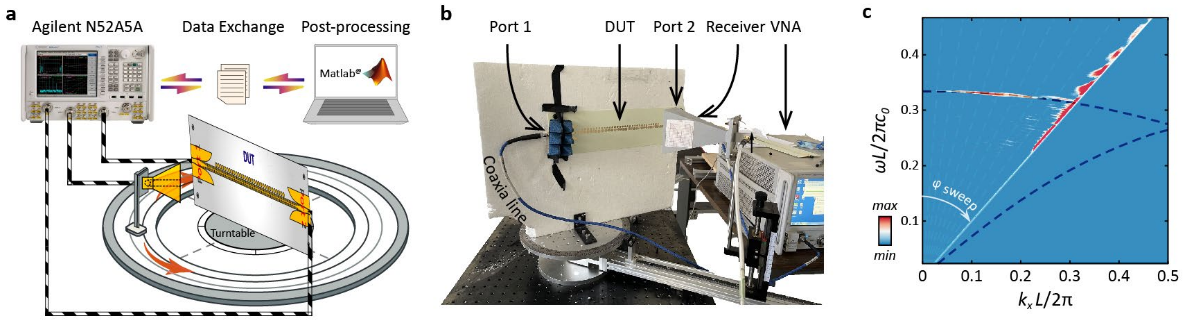

Figure S5. Illustration of the beam-scanning validation experiment. (a) Schematic diagram of the experimental setup; (b) Real experimental setup; (c) Measured evolutions of the S_{21} spectra versus radiation angle φ where φ is remapped into wavenumber space.

References

[1] T. Van Mechelen, Z. Jacob, Optica 2016, 3, 118.

[2] H. F. Ma, X. Shen, Q. Cheng, W. X. Jiang, T. J. Cui, Laser & Photonics Reviews 2014, 8, 146.
